# Dietary resistant starch ameliorating lipopolysaccharide-induced inflammation in meat ducks associated with the alteration in gut microbiome and glucagon-like peptide 1 signaling

**DOI:** 10.1186/s40104-022-00735-x

**Published:** 2022-07-15

**Authors:** Simeng Qin, Weiqiang Bai, Todd J. Applegate, Keying Zhang, Gang Tian, Xuemei Ding, Shiping Bai, Jianping Wang, Li Lv, Huanwei Peng, Yue Xuan, Quifeng Zeng

**Affiliations:** 1 Institute of Animal Nutrition, Key Laboratory for Animal Disease-Resistance Nutrition of China, Ministry of Education, Sichuan Agricultural University, Chengdu, 611130 China; 2 Department of Poultry Science, University of Georgia, 110 Cedar St, Athens, GA 30602 USA

**Keywords:** GLP-1, Inflammatory response, Intestinal integrity, Microbiota, Resistant starch

## Abstract

**Background:**

Consumption of resistant starch (RS) has been associated with various intestinal and systemic health benefits, but knowledge of its effects on intestinal health and inflammatory response in stressed birds is limited. Here, we examined how dietary RS supplementation from 12% raw potato starch (RPS) modulated inflammatory severity induced by lipopolysaccharide (LPS) in meat ducks.

**Results:**

LPS administration at 14, 16, and 18 d (chronic challenge) decreased body weight (BW) and glucagon-like peptide 1 receptor (GLP-1R) level with higher intestinal permeability and inflammation, evident by higher pro-inflammatory cytokine levels. Dietary 12% RPS supplementation enhanced Claudin-1 and GLP-1R expression, along with lower levels of inflammatory factors in both ileum and serum. Microbiome analysis showed that RS treatment shifted microbial structure reflected by enriched the proportion of Firmicutes, *Bifidobacterium*, *Ruminococcus*, etc. Dietary RS addition also significantly increased the concentrations of propionate and butyrate during chronic LPS challenge. Furthermore, response to acute challenge, the ducks received 2 mg/kg BW LPS at 14 d had higher concentrations of serum endotoxins and inflammatory cytokines, as well as upregulated transcription of toll like receptor 4 (*TLR4*) in ileum when compared to control birds. Analogous to GLP-1 agonist liraglutide, dietary RS addition decreased endotoxins and inflammation cytokines, whereas it upregulated the GLP-1 synthesis related genes expression. Meanwhile, dietary RS supplementation suppressed the acute LPS challenge-induced *TLR4* transcription.

**Conclusions:**

These data suggest that dietary 12% RPS supplementation could attenuate the LPS-induced inflammation as well as intestinal injury of meat ducks, which might involve in the alteration in gut microbiota, SCFAs production and the signaling pathways of TLR4 and GLP-1/GLP-1R.

**Supplementary Information:**

The online version contains supplementary material available at 10.1186/s40104-022-00735-x.

## Introduction

The intestine of animals and humans serves as the bridge for connecting intraluminal and enteric external milieu, which is not only responsible for digestion and absorption of nutrients, but also functions to prevent pathogenic entities invasion. During normal physiological activities, some endotoxins and toxins produced by colonized bacterial in intestine usually can be degraded and cleaved to non-toxic fragments by innate immune cells [1, 2]. Nevertheless, once the organism stuck in pathological conditions, the interrupted intestinal wall allow the pathogenic bacteria to tissue, the specific molecular patterns in microbial components could been recognized by some immune factors such as toll like receptors (TLRs) and induce the transcription of specific genes involved in pro-inflammatory and anti-bacterial responses [3]. These dysregulated immune responses due to intestinal disequilibrium may contribute to the occurrence of inflammatory related diseases including inflammatory bowel disease, sepsis, endotoxemia [4–6]. Modern meat ducks are no exception, various factors especially genetic selective pressure, environmental stress, bacterial infection, and immunological stress result in increasingly common incidence in the intestinal inflammation of birds [7]. The destroyed gut barrier integrity and dysbacteriosis could induce the systemic and intestinal inflammation [8]. In practically the early stage of growth and intestinal development, which is an important period relative to optimizing digestive efficiency and performance [7], the disorder of intestinal homeostasis in this phase has been noticed to associated with irreversibly weakened growth performance. Therefore, enhancing gut barrier integrity and reducing inflammation that are supposed to be an effective way for animal growth and physical health maintenance.

Currently, some functional feed ingredients targeting gut microbiota are acknowledged to counteract intestinal inflammation [9] and might be alternatives for antibiotics to product the antibiotic-free animal production in domestic birds. Resistant starch (RS), a kind of natural prebiotics exists in most common feed ingredients, is quite suitable for being supplemented in diets to maintain animal health. The most typical beneficial functions exerted by dietary RS is to enhance short chain fatty acids (SCFAs) production through optimizing gut microbiota in hindgut because of its relatively resistant to digestion in small intestine by starch degradation enzyme produced by host [10, 11]. Accumulating evidence from rodent studies indicated that RS supplemented diet were likely to alleviate tissue damage, including but not limited to intestine, during inflammatory stress by modulating inflammatory cytokines and gut microbiota [12–15]. However, the effect of RS on poultry was inconsistent. Recent research in broiler showed that the diets supplemented 4–12% corn RS could suppress intestinal morphology and barrier functions in jejunum [16]. On the contrary, data from broiler birds fed 5% butyralated high-amylose maize starch showed that RS could increase jejunal villus height/crypt depth ratios at 15 d and butyrate levels in both ileum and cecum at 15 and 24 d [17]. In line with this view, our previous studies showed that diet with 12% raw potato starch (RPS, Type II RS) could improve intestinal morphology and enhance intestinal barrier function through upregulating the transcription of tight junction proteins (TJPs) in both ileum and cecum, as well as it also increased the SCFAs production, gut microbial diversity and the abundance of beneficial bacteria including *Fecalibacterium* and *Subdoligranulum* in cecum of meat ducks [18, 19]. In addition to the metabolites such as SCFAs, glucagon-like peptide-1 (GLP-1) secretion might be a key contributor to the beneficial effects on the gut [20]. GLP-1 is a gut peptide secreted in enteroendocrine L cells located predominantly in the ileum and colon. It has been shown to against intestinal injury and promote gut growth via binding its receptor (GLP-1R) [21, 22]. Activation of GLP-1R is also associated with anti-inflammatory effects [23, 24]. It is reasonable to assume that dietary RS could alleviate intestinal inflammation from LPS challenge through GLP-1/GLP-1R signaling in meat ducks.

The objective of the current study, therefore, was to investigate the effects of dietary RS supplementation in the form of RPS on systemic and intestinal inflammatory response in chronic and acute lipopolysaccharide (LPS)-challenged meat ducks. Conceptually, acute injection of LPS corresponds broadly with acute, rapidly progressive sepsis, which is a well-recognized situation, subclinical infection followed by acute exacerbations is more common and associated with adverse outcomes. In addition, a peripheral inflammatory process must be considered a risk factor in the progression of intestinal injury. We thus decided to administer low doses of LPS in order to trigger the alert status of intestine, but avoiding a massive influx of pro-inflammatory cells to the gut, in an attempt to mimic a mild chronic peripheral condition. In the present study, intestinal barrier integrity, gut microbiota as well as the SCFAs production were assessed in chronic LPS-challenge model, and GLP-1/GLP-1R signaling as potential mechanism underlying the relationship between the improved intestinal status and dietary RS manipulation was also evaluated in acute LPS-challenge model. Our hypothesis was that dietary RS supplementation would protect against LPS-induced intestinal damage and abnormal release of inflammatory cytokines of meat ducks through SCFAs production and GLP-1/GLP-1R signaling under immunological stress.

## Materials and methods

The RS was used from RPS with a 57% RS content (Windmill® potato starch, Meelunie B.V., Holland). LPS was purchased from Sigma-Aldrich (From *Escherichia coli* serotype O55: B5; Sigma Aldrich L2637, MO, USA). GLP-1R agonist, liraglutide (Selleck, S8256, TX, USA). Birds were raised in temperature- and humidity-controlled room, and allowed to access water and feed freely throughout experiments. The male ducklings were used and vaccinated at 1 day of age against Newcastle Disease and Infectious Bronchitis at the hatchery facilities. The basal diet was formulated to meet the requirement of meat ducks according to National Research Council (NRC, 1994) [25]. All diets were isonitrogenous and isoenergetic and offered in pellet form (Additional file 1: Table S1–2). RS diet contained 12% RPS based on our previous work [18].

### Birds and study design

To evaluate the response of intestinal status to chronic or acute LPS challenge and dietary RS supplementation. In the chronic LPS challenge, 240 1-day-old Cherry Valley male meat ducks were randomly assigned to 3 treatments in 8 replicate pens (10 birds per pen): the control group (basal diet), LPS challenge (LPS) group (basal diet), and LPS challenge with fed 12% RPS diet (LPS + RS) group (RS diet). After 12-h feed deprivation, the ducks were intraperitoneally injected with either 1 mg/kg body weight (BW) of LPS or sterile saline at 14, 16, and 18 d (Fig. S1A). The routes of LPS injection were identical to our previous study [26]. Four hours after injecting on 18 d, 1 duck with a weight closest to the pen average was selected for sampling. Blood samples were collected and centrifuged at 3000 × *g* for 15 min at 4 °C to gain serum for endotoxins and inflammatory cytokines contents test, and subsequently the distal ileum and ileal mucosa were obtained for histological analysis and gene expression. In addition, cecal digesta were collected for SCFAs determination and microbial analysis.

For the acute LPS challenge, a total of 144 1-day-old Cherry Valley male meat ducks were randomly assigned to 4 treatments in 6 replicate pens (6 birds per pen): the control group (basal diet), LPS (basal diet), LPS + RS (12% RPS diet), or LPS challenge with liraglutide (100 μg/kg BW; LPS + Liraglutide) group (basal diet), respectively. At d 14, birds were injected intraperitoneally with either 2 mg/kg BW of LPS or sterile saline (Fig. S1B). At 4 h after injecting, 1 bird from each pen was selected. Blood was taken to obtain serum for endotoxins and inflammatory cytokines contents test or plasma for GLP-1 content analysis, and then distal ileum and ileal mucosa were collected for histological analysis and gene expression, respectively.

### Histological analysis

The fixed distal ileum using 10% neutral-buffered formalin for 24 h was embedded in paraffin. Sections of 4-μm thickness were subjected to hematoxylin and eosin staining (H&E). Images were taken from a microscope (BA400Digital, Mike Audi Industrial Group Co., Ltd., China) and were analyzed with Image-Pro Plus 6.0 (Media Cybernetics, Silver Spring, MD, USA).

### Immunofluorescence analysis

The GLP-1R protein of ileum was determined by immunofluorescence. The 4% paraformaldehyde-fixed samples were rehydrated in PBS, subjected to antigen retrieval with ethylene diamine tetraacetic acid buffer, and then blocked with 3% bovine serum albumin prior to incubation with mouse monoclonal antibodies to GLP-1R (1:100; sc-390774; Santa Cruz Biotechnology Inc., Dallas, TX, USA) overnight at 4 °C. Slides were then detected with Cy3 conjugated Goat Anti-mouse IgG (GB21301; Servicebio Technology Co., Ltd., Wuhan, China) for 1 h at room temperature in the dark, and the nuclei were stained with 4′-6-diamidino-2-phenylindole (DAPI) for 10 min. All slides were finally examined for fluorescence using a confocal scanning microscope (NIKON ECLIPSE TI-SR), and images were taken with the NIKON DS-U3 software. The proportion of GLP-1R positive cells was determined by counting cells in each of three sections from each of 6 ducks.

### Intestinal permeability determination

Ducks from chronic LPS challenge trial were used to determinize the intestinal permeability using fluorescein isothiocyanate dextran (FITC-d, 4 kDa, Sigma, USA), an indicator to examine barrier function. On d 18, 1 bird each pen close to the pen average weight were selected, and all ducks received orally FITC-d (4.16 mg/kg). The serum was collected at 2.5 h post FITC-d administration and the fluorescence was measured at 485 nm excitation and 528 nm emission (BioTek Instruments, Winooski, VT). The FITC-d concentrations were determined from standard curves generated by the serial dilution of FITC-d.

### Measurement of GLP-1 in plasma and cytokines in serum and ileum

Cytokines of interleukin-1β (IL-1β), IL-6, IL-17, and tumor necrosis factor-α (TNF-α) or IL-4 concentrations in serum and ileal mucosa, as well as GLP-1 concentration in plasma were measured using enzyme-linked immunosorbent assay (Meimian Biotechnology Co., Ltd., Jiangsu, China). Endotoxins concentration was analyzed by a commercially available Chromogenic End-point Tachypleus Amebocyte Lysate (CE TAL) kit (Nanjing Jiancheng Bio-Engineering Institute Co., Ltd., Nanjing, China) and expressed as EU/mL. All assays were performed as described by the manufacturer’s instructions and done in duplicate.

### Gene expression assays

Total RNA was extracted using a Trizol reagent (TaKaRa, China) from frozen ileal mucosa samples following the manufacturer’s instructions. RNA integrity was tested by a 1% agarose gel electrophoresis. Real-time PCR was performed on ABI QuantStudio™ 6 Flex system (Applied Biosystems, CA, USA). The primer sequences for the target genes were designed using Primer 3 (Additional file 1: Table S3). Relative gene expression was quantified by normalizing to the expression of glyceraldehyde-3-phosphate dehydrogenase (*GAPDH*) and β-actin.

### Cecal SCFAs analysis

Approximately 0.5 g of cecal digesta was diluted with 2 mL of ultrapure water mixed with a uniform. The solution was left for 30 min and then centrifuged at 3000 × *g* for 15 min. Supernatants (1 mL) were mixed with 0.2 mL ice-cold 25% (w/v) metaphosphoric acid solution, and incubated at 4 °C for 30 min, followed by 11,000 × *g* centrifugation for 10 min. The SCFAs contents including acetate, propionate, and butyrate were separated and determined by gas chromatograph (Varian CP-3800, NJ, USA).

### Gut microbiome sequencing

Total DNA was extracted from cecal content with a DNA stool mini kit (Qiagen, Valencia, CA, United States). After determining the DNA concentration and integrity, an amplicon sequencing library was constructed based on the PCR-amplified V4 variable regions of 16S rDNA, and paired-end sequenced on an Illumina HiSeq 2500 platform (BGI, Shenzhen, China). The obtained sequences were processed using FLASH (v1.2.11) and USEARCH (v7.0.1090) for alignment and clustering. The final reads were clustered as operational taxonomic units (OTUs) with a 97% similarity threshold. The OTUs were further assigned to species level using the RDP classifer (v2.2) based on the Greengene database (v201305) of full-length 16S rRNA sequences. Partial least-squares discrimination analysis (PLS-DA) was performed by R package mixOmics.

### Statistical analysis

The data obtained were analyzed by the Shapiro-Wilk and Levene’s test to assess normal distribution and homogeneity of variances. Analyses were done using one-way analysis of variance (ANOVA) followed by Tukey’s test for multiple comparisons and the Mann-Whitney U test for normally or non-normally distributed datasets using SAS 9.2 (SAS Institute, Inc.), respectively. For data of microbiota, partial least square discriminant analysis (PLS-DA) was performed on genus data to identify the effects of treatments, in which *Y* is a set of variables describing the categories of a categorical variable on *X*, and *X* variables are bacterial genera and *Y* variables are experimental treatments. In this analysis, to avoid over parameterization of the model, variable influence on projection value (VIP) was estimated for each genus and genera with VIP < 0.50 were removed from the final model [27]. R^2^ estimate and Q^2^ estimate were used to evaluate the goodness of fit and the predictive value of the model, respectively. The PLS-regression coefficients were used to identify genera that were most characteristic of each treatment group, and the results were visualized by PLS-DA loading scatterplots. Statistical differences in relative bacterial abundances between treatments were tested by non-parametric Kruskal-Wallis tests with Tukey-Kramer *post-hoc* tests and the Benjamin-Hochberg false discovery rate using the R software (Version 3.4.1) on the genus levels. In addition, Spearman correlation analysis was used to explore the correlation between gut bacteria and gene expression of cytokines. Differences with *P* ≤ 0.05 were considered significant, and 0.05 < *P* < 0.10 to reflect a tendency.

## Results

### Dietary RS inclusion improved BW and intestinal barrier during chronic LPS challenge

**Fig. 1:**
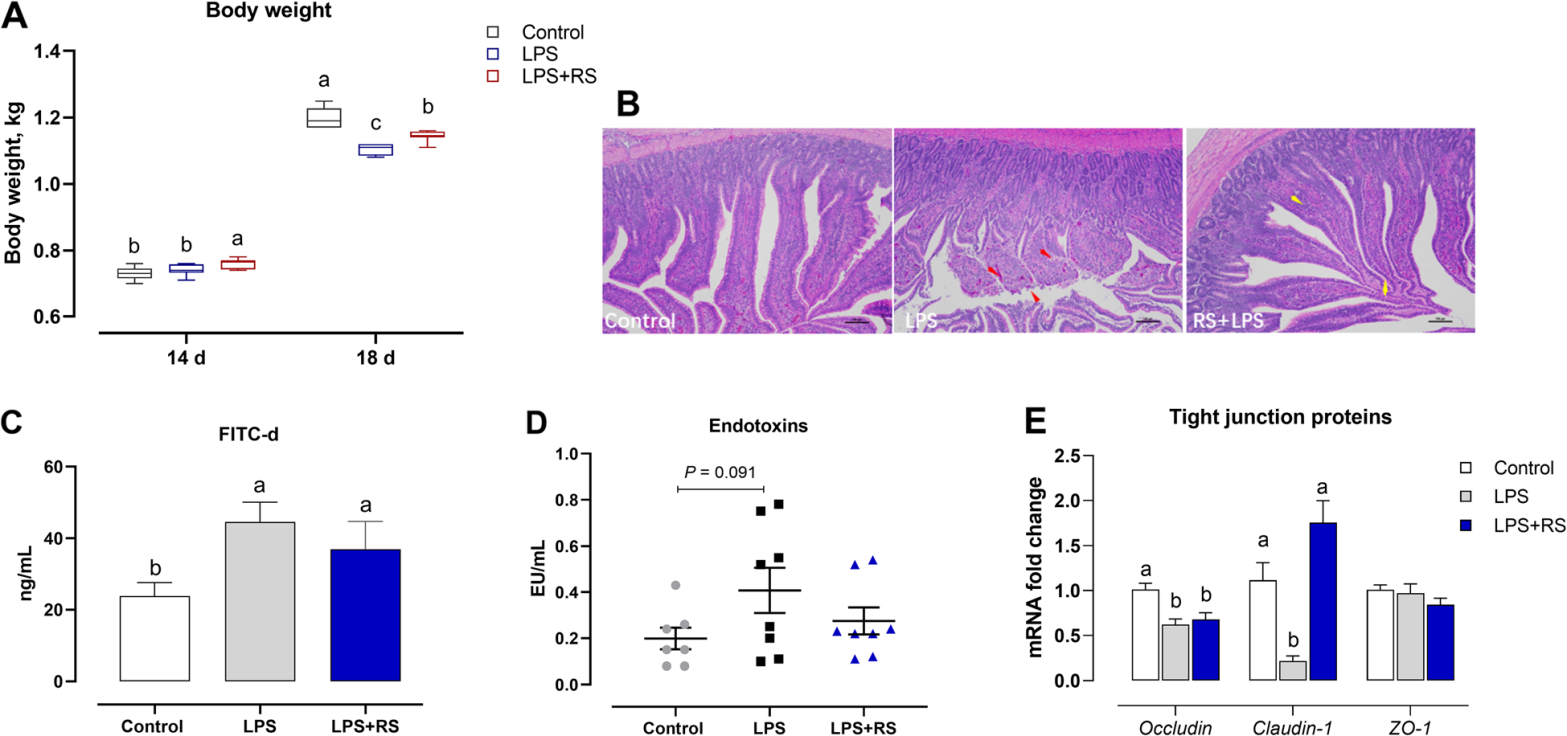
Dietary resistant starch (RS) reversed the chronic lipopolysaccharide (LPS)-induced the compromised body weight and the impaired intestinal barrier integrity. **A** Body weight; **B** Representative hematoxylin/eosin (H&E) staining of ileum; Gut permeability was evaluated by (**C**) Fluorescein isothiocyanate-dextran (FITC-d) and (**D**) Serum endotoxins; **E** The genes encoding tight junction proteins including zonula occludens-1 (*ZO-1*), *Occludin*, and *Claudin-1* in ileal mucosa were determined. ^a-c^Mean values with different letters are significantly different (one-way ANOVA, *P* ≤ 0.05, Tukey’s post hoc test)

Prior to LPS challenge, RS inclusion significantly increased (*P* < 0.05) ducks’ BW at 14 d. Subsequently, the BW at 18 d was notably decreased by chronic LPS-challenge, which was reversed due to dietary RS treatment (both *P* < 0.05; Fig. 1A). Regarding intestinal barrier, histologic examination showed that ileum form the control group presented a regularly arranged villi and intact epithelial structure (Fig. 1B). LPS injection resulted in obviously exfoliated ileal mucosa, disorderly arranged and broken villi and infiltrated with inflammatory cells, whereas the diet with RS alleviated ileal injury, showed by basically intact epithelia and slightly inflammatory cell infiltration after chronic LPS-challenge (Fig. 1B).

The outcomes from FITC-d and endotoxins, both biomarkers used to reflect the alteration of intestinal permeability, suggested that the concentrations of FITC-d (*P* < 0.05) and endotoxins (*P* = 0.091) in serum were elevated in chronic LPS-challenge group when compared with the control group, which was decreased by RS supplementation to varying degrees (*P* > 0.05; Fig. 1C, D). Moreover, the genes encoding intestinal barrier were determined and showed that chronic LPS challenge significantly decrease mRNA expression levels of *Occludin* and *Claudin-1* in ileum as compare with the control group, and the altered mRNA abundance of *Claudin-1* was remarkably reversed with dietary RS administration (*P* < 0.05; Fig. 1E).

### Gut microbiota and cecal SCFAs concentrations response to chronic LPS challenge and dietary RS supplementation

**Fig. 2:**
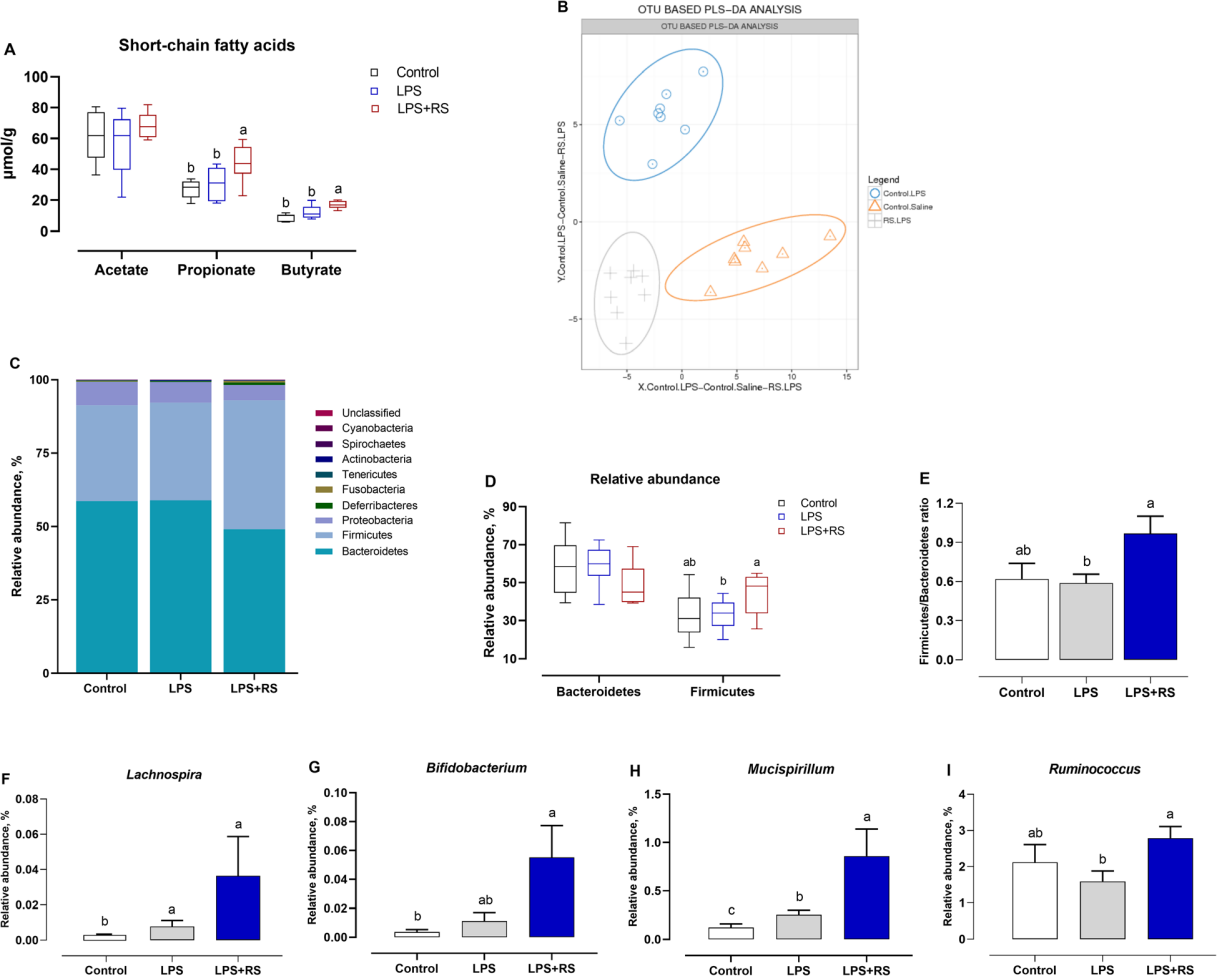
Short-chain fatty acids (SCFAs) and gut microbiome in response to dietary resistant starch (RS) supplementation under chronic lipopolysaccharide (LPS) challenge. **A** Concentrations of SCFAs in cecal contents; **B** Partial least-squares discrimination analysis (PLS-DA) analysis; **C** Composition profiles of gut microbiome at phyla level among treatments revealed by 16S rRNA sequencing; Alterations of relative abundance of representative bacteria (**D**) Bacteroidetes and Firmicutes, and (**E**) calculated the ratio of Firmicutes/Bacteroidetes, (**F**) *Lachnospira*, (**G**) *Bifidobacterium*, (**H**) *Mucispirillum*, (**I**) *Ruminococcus*. ^a-c^Mean values with different letters are significantly different

As illustrated in Fig. 2A, there were no significant changes in individual SCFAs by challenging LPS (*P* > 0.05). Dietary RS supplementation significantly increased the concentrations of propionate and butyrate during chronic LPS challenge (*P* < 0.05). With regarding to the gut microbiota, the composition was apparently affected by experiment manipulation, indicated by totally separated from treatments (Fig. 2B). More specifically, except increased the proportion of *Lachnospira* and *Mucispirillum*, the chronic LPS injection failed to change the abundance of Firmicutes, Bacteroides, *Bifidobacterium*, and *Ruminococcus* compared to control group. However, diet with RS increased the proportion of Firmicutes, and thus increased the ratio of Firmicutes to Bacteroides, along with higher abundance of *Bifidobacterium*, *Mucispirillum*, and *Ruminococcus* following the LPS-challenge (Fig. 2C-I).

### Dietary RS treatment alleviated chronic LPS challenge-induced inflammatory reaction, which might be relevant to the alteration in gut microbiota and GLP-1 signaling pathway

**Fig. 3:**
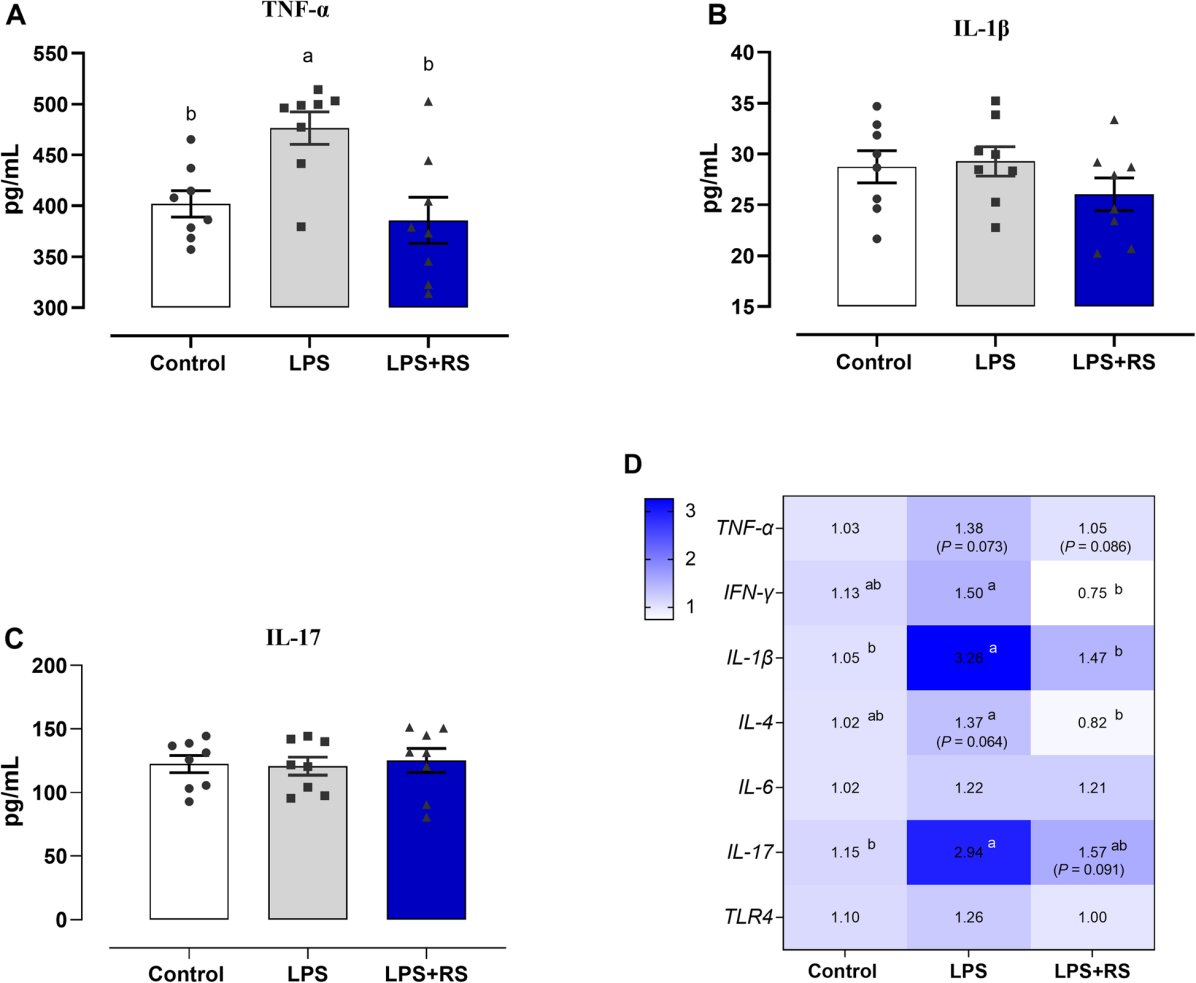
Dietary resistant starch (RS) ameliorated the chronic lipopolysaccharide (LPS)-induced systemic and intestinal inflammation. Inflammatory cytokines (**A**) TNF-α, (**B**) IL-1β and (**C**) IL-17 concentrations in serum. **D** A heat map showing the mRNA expression of inflammatory cytokines in ileum. IL, interleukin; TNF, tumor necrosis factor; IFN, interferon; TLR, toll like receptor. ^a,b^Mean values with different letters are significantly different (one-way ANOVA, *P* ≤ 0.05, Tukey’s post hoc test)

Concerned the ileal and systemic inflammatory response, LPS challenge induced higher concentration of serum TNF-α relative to control group, which was normalized by supplementing dietary RS (*P* < 0.05; Fig. 3A). However, experiment treatments did not alter the content of IL-1β and IL-17 in serum (*P* > 0.05; Fig. 3B, C). Regarding the ileum, the mRNA levels of *TNF-α* (*P* = 0.073), interferon-γ (*IFN-γ*), *IL-1β*, *IL-4* (*P* = 0.064) and *IL-17* were increased in chronic LPS administration (*P* < 0.05), and reversed their increased levels by dietary RS (Fig. 3D).

**Fig. 4:**
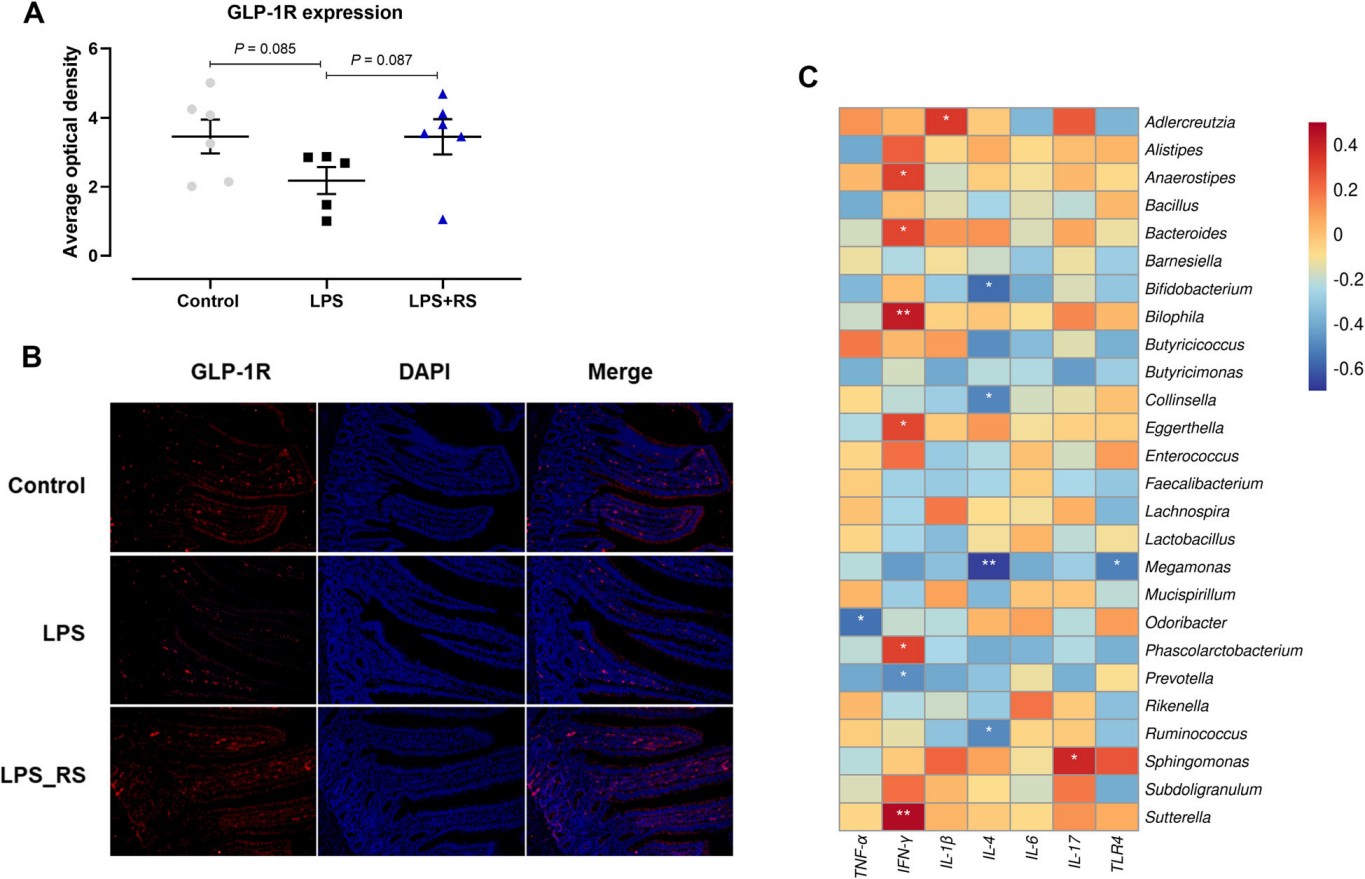
**A** Expression and (**B**) distribution of glucagon-like peptide-1 receptor (GLP-1R) expression response to dietary resistant starch (RS) administration under chronic lipopolysaccharide (LPS) challenge were assessed using immunofluorescence (IF) staining. **C** Spearman correlation analysis of the cecal microbiota and ileal inflammatory cytokines gene expression. *P*-values the letter * are significant correlation (*P < 0.05; **P < 0.01) and depicted from blue to red, where red represents a positive correlation and blue represents a negative correlation

We further analyzed the potential association between inflammatory response, GLP-1 signaling, and gut microbiota. The result of immunofluorescence analysis showed that LPS challenge tended to decrease the GLP-1R expression (*P* = 0.085), and then the diets supplemented with RS upregulated the abundance of GLP-1R in ileum (*P* = 0.087; Fig. 4A, B). In addition, alterations in bacteria were also associated with one or more inflammatory response factors based on Spearman’s correlation coefficients (Fig. 4C). For instance, at genera levels, both *IL-4* and *TLR4* were negatively correlated with *Megamonas*. *IL-4* was also significantly negatively correlated with some health-associated gut bacteria, including *Bifidobacterium*, *Collinsella* and *Ruminococcus*. *TNF-α* was negatively associated with the abundance of *Odribacter*. *IL-1β* and *IL-17* are positively associated with *Adlercreutzia* and *Sphingomonas*, respectively. *IFN-γ* associated positively with *Anaerostipes*, *Bacteroides*, *Eggerthella* and *Phascolarctobacterium*, and most notably *Sutterella* and *Bilophila*, while negatively with *Prevotella*.

### Dietary RS improved ileal integrity and decreased inflammation response during acute LPS challenge

**Fig. 5:**
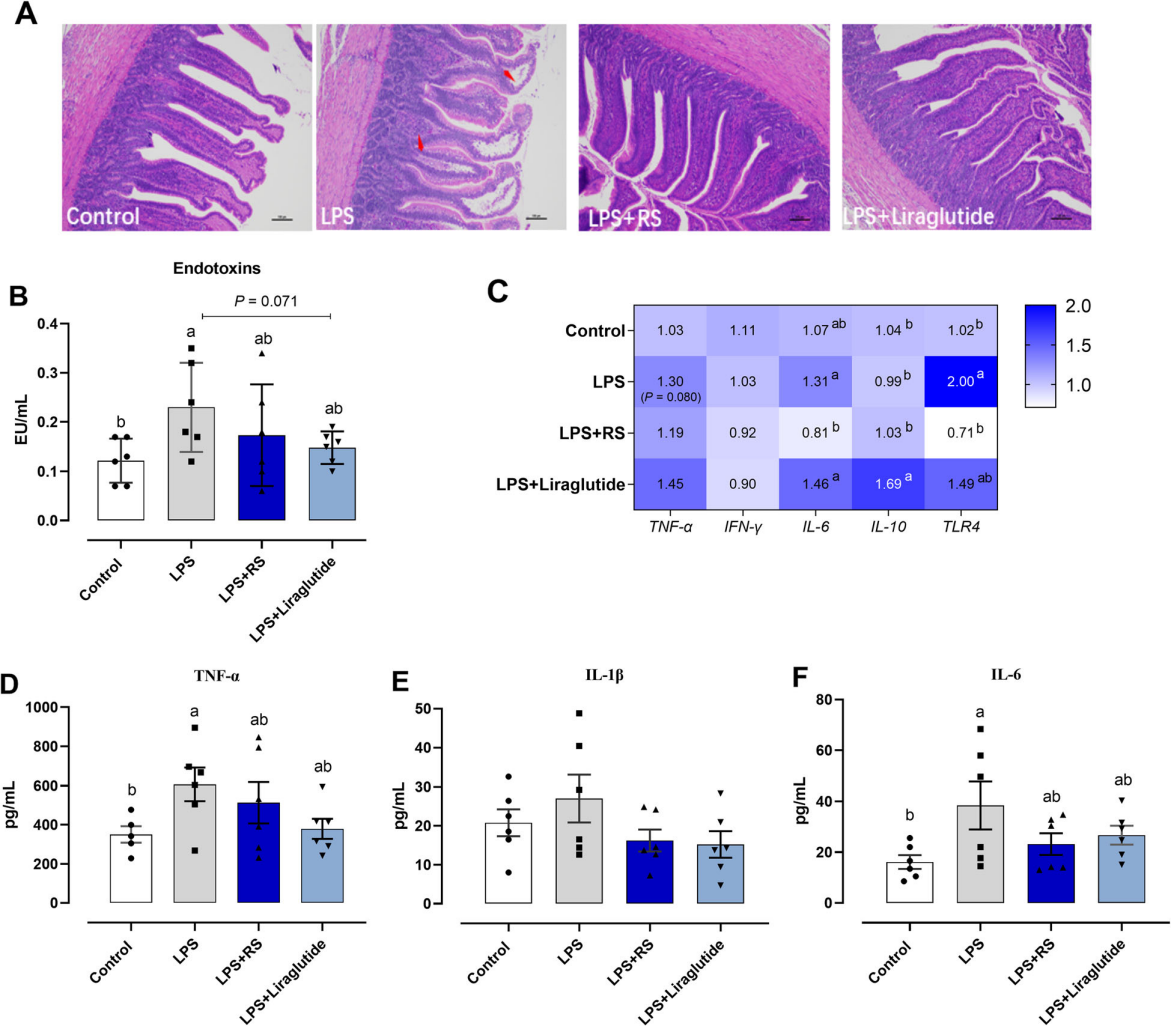
Dietary resistant starch (RS) and liraglutide administration ameliorated the acute lipopolysaccharide (LPS)-induced systemic and intestinal inflammation and the compromised intestinal permeability. **A** Representative hematoxylin/eosin (H&E) staining of ileum; **B** Gut permeability was evaluated by serum endotoxins; **C** A heat map showing the mRNA expression of inflammatory cytokines in ileum. IL, interleukin; TNF, tumor necrosis factor; IFN, interferon; TLR, toll like receptor. Inflammatory cytokines; **D** TNF-α, (**E**) IL-1β and (**F**) IL-6 concentrations in serum. ^a,b^Mean values with different letters are significantly different (one-way ANOVA, *P* ≤ 0.05, Tukey’s post hoc test)

To further explore the role of GLP1/GLP-1R signaling in the beneficial effect of dietary RS attenuating intestinal inflammation induced by LPS challenge. The model of acute LPS challenge and GLP-1R agonist liraglutide were used, and the results showed that dietary RS inclusion did not significantly influence the BW in acute LPS challenged ducks among treatments (Fig. S1C). Histologic examination revealed that ileum of ducks in control groups presented a regularly arranged villi and intact epithelial structure, However, heavily infiltrated with inflammatory cells and partially exfoliated ileal mucosa were observed in LPS group, which were mitigated by RS supplementation or liraglutide injection, evident by basically intact epithelia with slightly inflammatory cell infiltration after acute LPS-challenge (Fig. 5A). The higher serum endotoxins level suggested an increase in gut permeability due to acute LPS challenge group when compared with that in control (*P* < 0.05). Both liraglutide injection and dietary RS supplementation reduced the concentration of serum endotoxins to varying degrees (Fig. 5B).

Moreover, acute LPS challenge also elevated genes expression of *TNF-α* (*P* = 0.080) and *TLR4* (*P* < 0.05) in ileum as compared to the control, and dietary RS supplementation markedly reversed *TLR4* mRNA level (Fig. 5C). Meanwhile, dietary RS and liraglutide treatment were significantly lowered *IL-6* or promoted *IL-10* transcriptional levels (both *P* < 0.05) relative to LPS-challenge group (Fig. 5C). In serum, acute LPS challenge increased the concentration of TNF-α and IL-6 as compared with control group (*P* < 0.05), and subsequently were suppressed by the administration with liraglutide injection or dietary RS (*P* > 0.05; Fig. 5D-F).

### GLP-1/GLP-1R signaling responses to dietary RS addition during acute LPS challenge

**Fig. 6:**
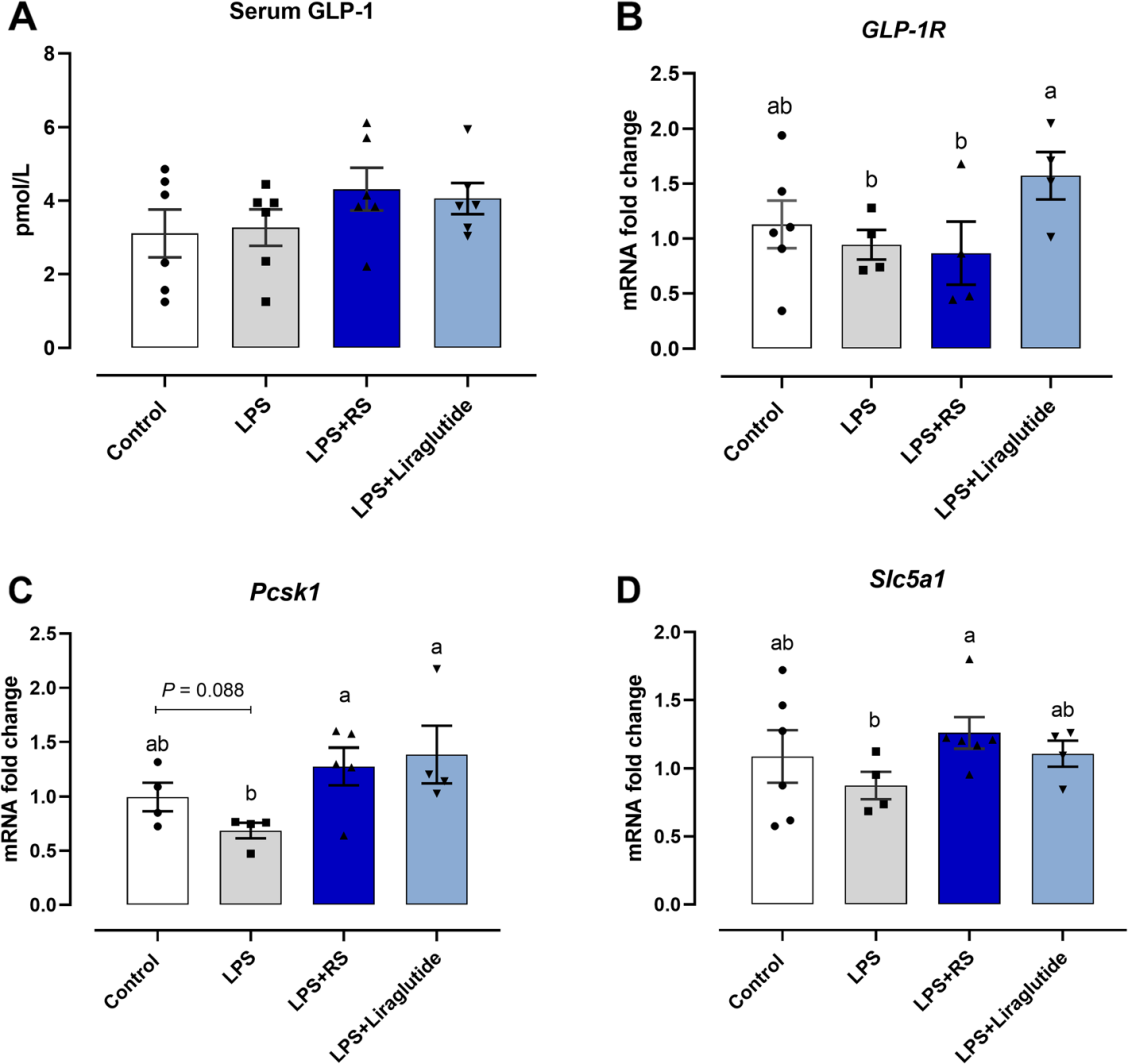
Glucagon-like peptide (GLP) -1 signaling response to dietary resistant starch (RS) and liraglutide administration under acute lipopolysaccharide (LPS) challenge. **A** Plasma GLP-1 contents; Genes expression of (**B**) *GLP-1R*, (**C**) proprotein convertase subtilisin/kexin type (*Pcsk1*) and (**D**) solute carrier family 5 member 1 (*Slc5al*). ^a,b^Mean values with different letters are significantly different (one-way ANOVA, *P* ≤ 0.05, Tukey’s post hoc test)

As presented in Fig. 6A, although acute LPS change did not significantly change the concentration of plasma GLP-1, administrated with dietary RS and liraglutide result in a 32% and 24% increase in terms of serum GLP-1 content compared with LPS group, respectively (Fig. 6A). The GLP-1 synthesis related genes expression including proprotein convertase subtilisin/kexin type (*Pcsk1*) and solute carrier family 5 member 1 (*Slc5al*), as well as GLP-1R were also determined, and the data suggested that acute LPS challenge did not notably affect the GLP-1R and *Slc5al* genes expression, but tended to increase the abundance of *Pcsk1* mRNA (*P* = 0.088; Fig. 6B-D). When compared to LPS group, liraglutide injection increased *GLP-1R* and *Pcsk1* mRNA level (*P* < 0.05; Fig. 6B, C). Also, the diet supplemented with RS upregulated that transcription of *Pcsk1* in ileum (*P* < 0.05; Fig. 6C, D).

## Discussion

Intestinal inflammatory disease has been becoming a great threat to poultry production in non-obvious ways due to impaired gut and growth performance of birds [28, 29]. Numerous researches that included our previous study have confirmed the release of pro-inflammatory cytokines and immunological stress triggered by LPS intraperitoneal injection could impair nutrients absorption and depress growth performance in domestic birds [26, 30, 31]. Considered the rapid alteration of general homoeostasis caused by LPS [2] and the retardance of disturbances in microbiome composition within a single day of initiating a diet [32], an intermittently and repeatedly intraperitoneal injection of low doses of LPS into birds was used as a chronic LPS-induced immunological stress model, so that RS has an extended period to exert its potential benefits through modulating gut microbiota in response to LPS challenge. In the present study, the reduced BW by chronic LPS injection has yielded strong evidence for a harmful consequence on growth performance of meat ducks induced by LPS challenge. Furthermore, the compromised BW by chronic-LPS challenge at 18-d-old ducks were elevated by RS supplementation, indicating that RS exerted protective effects on performance of ducks suffered from immunological stress. Our previous study noticed that RS functioned as prebiotics to maintain intestinal health of ducks by improving barrier function and modulating microbial composition [18, 19], which could explain why the dietary RS ameliorated LPS-induced compromised growth performance in LPS-challenge ducks. For this, intestinal morphology and permeability were further determined in the currently study. In lined with previous findings in ducks [31], the higher serum FITC-d concentration suggested that LPS challenge resulted in a drastic increase intestinal permeability. At the molecular level, chronic LPS challenge downregulated the expression of *Claudin-1* and *Occludin*, both important composition of TJPs in intestinal barrier [33]. Evidence from our recently studies showed that dietary RS administration could enhanced gut barrier function of ducks in normal feeding via increasing mRNA expression of TJPs in ileum [18]. In addition, RS was also found to effective in protecting the TJPs expression in dextran sulfate sodium (DSS)-induced mice [13]. The present results are congruent with previous outcomes and indicated that dietary RS is beneficial to enhance intestinal barrier integrity under immunological tress.

It is well-known that compromised intestinal integrity might trigger aberrant inflammatory responses and in conjunction with accumulation of inflammatory mediators and further aggravating tissue damage [34]. Herein, we also compared the differences in alteration of systemic and intestinal inflammation response to LPS challenge and dietary RS supplementation. In this study, inflammatory lesions in ileum was consistently observed in LPS-challenge ducks, and dietary RS supplementation could effectively alleviate inflammatory injury in villus and crypt of ileum induced by LPS. RS provided also exerted a protective role against DSS-induced colonic inflammation as reflected by a decrease in colonic pro-inflammatory cytokine levels of IL-1β, IL-6, TNF-α, and IFN-γ in proximal or distal segments [13]. Analogously, our results in ileum showed that chronic LPS-challenge increased the expression of *IL-1β*, *IL4*, *IL-17*, and *TNF-α*, and acute LPS challenge upregulated the *TNF-α* mRNA levels. As an anti-inflammatory effect, dietary RS supplementation alleviated inflammatory response in ileum both in acute and chronic LPS challenge, evidenced by a noticeable reduction in transcriptional levels of those increased inflammatory cytokines mentioned above. Of note, the lipid A fraction contained in LPS can act as ligand for immune receptor TLR4 that elicits robust inflammatory responses in immune and somatic cells [35]. TLR4 is required to recruit specific adaptor proteins including myeloid differentiation primary response gene etc., to initiate the downstream signaling to produce inflammatory cytokines [36] and the activation of TLR4 is preceded the release of inflammatory cytokines [37]. TLR4 is thus considered as a classical signaling pathway in triggering cytokine production, inflammation and adaptive immune response. Whereupon, the gene expression of *TLR4* in ileum was examined to verify whether RS exerts an anti-inflammatory effect through this pathway. The present study with RS supplementation observed a remarkably reversion in LPS-induced increase in *TLR4* mRNA level under acute LPS challenge. However, this result failed to observe in chronic LPS challenge, which might be caused by the repetitive injection of LPS into ducks, to some degree, induced a state of LPS tolerance that subsequently diminished TLR4 signaling [38]. In spite of this, our results still support the ability of dietary RS to exhibited an excellent anti-inflammatory efficacy when suffered LPS challenge, which might involve in the suppression of TLR4 role.

Reflecting to systemic inflammation, our outcomes from serum inflammatory cytokines showed that both acute and chronic LPS challenge increased concentrations of serum endotoxins. In addition to as indictor of intestinal permeability, endotoxin is also a sort of LPS that constituting much of the outer membrane of gram-negative bacteria. When suffered bacterial infection, high concentrations of endotoxins in gut and many other tissues would be present [39]. According to results of researches in human subjects, endotoxins levels in plasma or serum are normally low, but elevated during infections or gut inflammation [35]. Therefore, systemic inflammation caused by LPS injection in this study may be evidenced by the elevated endotoxins in serum of ducks. Meanwhile, LPS induces inflammation indirectly via the pro-inflammatory cytokines such as TNF-α, IL-6 and IL-1β was related to increased endotoxins level [39]. We therefore examined a variety of serum inflammatory cytokines concentrations in the current study. The concentration of TNF-α significantly increased after chronic LPS-challenge and both the content of TNF-α and IL-6 in serum were also stimulated by acute LPS-challenge, which were reduced by dietary RS supplementation to varying degrees, implying that dietary RS supplementation contributes to normalized the release of pro-inflammatory cytokines during inflammatory infection induced by LPS challenge.

Gut microbiota is considered to be a crucial part of intestinal homeostasis and paly key role in inflammatory response. There are accumulating evidence have confirmed that RS is beneficial for modulating microbiota in hindgut [10]. As we described in our previous study, diet with 12% RPS had a higher relative abundance of Firmicutes [19]. In the present study, we also confirmed that RS supplementation resulted in a shift in the microbial structure, and there was a consistent increase in the abundance of Firmicutes and the ratio of Firmicutes and Bacteroidetes following RS diet with LPS-challenge compared to basal diet with LPS-challenge, suggesting that members of the Firmicutes had more selective advantage than members of the Bacteroides when supplementing diet with RS, as similar results were also observed in study on human with high levels of RS diet [11]. *Ruminococcus brommi* that belongs to *Ruminococcus* has been shown to degrade RS, and numerous studies in human and mice have reported RS supplementation could increase the amounts of *Ruminococcus* and *Lachnospira* [11, 15, 40, 41]. Besides, following these changes, the relative abundance of *Ruminococcus* and *Lachnospira* were enriched in RS supplemented diet. *Lachnospira* is known as butyrate-producing bacteria [42]. *Ruminococcus brommi* is regarded as a major taxon to provide fermentation substrates and increase acetate concentrations for the growth of various major butyrate producers exhibiting the butyrate transferase, which leading to increased concentrations of the SCFAs, specifically butyrate [43]. These pieces of evidence supported that the increased their abundance was in concert with the increased butyrate in the cecal contents of ducks consuming RS diet. In addition, SCFAs is known as the microbiota-derived metabolites produced in cecum and colon, which are the signatures of the gut microbiota and contribute to modulate intestinal immune activity and inflammatory responses [44]. Our results of SCFAs showed that dietary RS supplementation elevated the concentration of propionate and butyrate, which was in agreement with our previous study [19], in which diet supplemented with 12% RPS could increase SCFAs contents in normal feeding experiment. To link the gut microbiota with the inflammatory markers, a correlation analysis was integrated and suggested that there were various bacteria significantly associated with these inflammatory cytokines and mediators. For example, the RS-degrading organisms *Bifidobacterium* and *Ruminococcus* were negatively associations with *IL-4*. *Bilophila* was positively related to *IFNγ*. Pathobiont *Bilophila* was associated with aggravates metabolic dysfunctions in mice [45]. These results indicated that alterations in cecal microbial composition derived by dietary RS supplementation may play a pivotal role in alleviating inflammatory responses induced by LPS challenge.

Emerging evidence demonstrated that inflammatory stimuli increased the GLP-1 secretion, which was found increased in rodents with experimental inflammation induced by LPS challenge [23, 46, 47]. In turn, GLP-1 could modulate inflammation in multiple sites such as blood vessels [48]. Considering the inseparable relations between dietary RS treatment and GLP-1 secretion [20]. The GLP-1R agonist liraglutide was used to dig the role of RS in inflammatory response through GLP-1 production. GLP-1 exerts anti-inflammatory capacities through directly reduce inflammation in organs expressing the GLP-1R, and/or by GLP-1 targets GLP-1R expressed on circulating immune cells indirectly reduce inflammation [48]. Natheless, GLP-1 as a peptide hormone is limited by its short half-life in vivo due to rapid degradation by dipeptidyl peptidase IV (DPP-4) [49]. A single high-dose injection of LPS (acute LPS-challenge), therefore, was used as the inflammatory immunostimulating model to explore the potential GLP-1-associated mechanism. In the present study, dietary RS addition alleviated pro-inflammatory cytokine levels including TNF-α, IL-6 and IL-1β, which were similar to the anti-inflammatory effect exerted by liraglutide in serum. However, the downregulated mRNA abundance of *IL-6* and *TLR4* and comparable anti-inflammatory cytokine *IL-10* transcription by dietary RS administration was incompatible with the role of liraglutide. To further illustrate this, serum GLP-1 content and the GLP-1 synthesis related genes including *Pcsk1* and *Slc5al* were determined in the present study. The data showed that GLP-1 secretion was consistently increased in dietary RS supplementation and liraglutide administration during acute LPS challenge, and the diet supplemented with RS upregulated the transcription of *Pcsk1*, which was analogous to it in liraglutide injection. Therefore, this improvement in intestinal and systemic inflammation from our data may indirectly reflect long-term RS supplementation plays as a stimulator in activation of GLP-1/GLP-1R signaling. Indeed, previous research showed that augmentation of various cytokines gene expression in the jejunum by continuously GLP-1R agonist Ex-4 treated was rapidly returning to normal by 24 h, which also indicated that GLP-1 could suppresses a pro-inflammatory cytokine program, and the action requiring a functional GLP-1R [24]. Collectively, our findings provide a new angle of view for regulating host and intestinal inflammation by dietary RS treatment through GLP-1/GLP-1R signaling.

This study raises 4 new questions need to be elaborated. The first is the response of gut microbiota to chronic LPS challenge. It was established that inflammation is triggered by LPS derived from the gut microbiota. However, in this study, except increased the proportion of *Lachnospira* and *Mucispirillum*, the chronic LPS injection failed to change the abundance of Firmicutes, Bacteroides, *Bifidobacterium*, and *Ruminococcus.* The second is the difference of GLP-1R expression between acute and chronic LPS challenge. Herein, chronic LPS challenge decreased the expression of GLP-1R using histometric method, whereas no apparently change was observed in acute LPS injection via RT-PCR determination. The third is LPS challenge model. The assessment for the link of dietary RS, inflammation, and GLP1/GLP-1R signaling was conducted using acute but not chronic LPS challenge, it is possible that the responses of GLP1/GLP-1R signaling to acute and chronic LPS challenge are complicated and changeable. The fourth is expression of IL-4 in ileum. In general, IL-4 is an anti-inflammatory interleukin and able to promote wound healing and tissue repair. However, some researches pointed out that the LPS-challenge resulted in higher the expression of *IL-4* mRNA expression in the duodenum of chicken [50]. In line with our studies, dietary 20% RS from high amylose maize starch was proved to reverse hepatic inflammation through depressing *IL-4* mRNA transcription in liver of mice fed high-fat diet [15]. Theses data implied that there might be both anti-inflammatory and pro-inflammatory roles exerted by IL-4 in process of RS ameliorating LPS-induced inflammation in meat ducks. Therefore, some of inaccurate conclusion highlighted the roles of dietary RS addition in modulating LPS-induced inflammatory response might be included in this study. Further research would be essential to exclude this possibility.

## Conclusion

In conclusion, 12% RPS supplementation reversed weight gain, inflammation, and increased intestinal permeability in meat ducks subjected to LPS-challenge. Furthermore, the ameliorative effects of RS supplementation were associated with altered microbiome and SCFAs production, as well as enhanced GLP-1 synthesis related genes and GLP-1R expression. These results will bring a valuable strategy of nutritional immunity for protecting host by exploiting dietary RS supplementation ways to against immunological stress for domestic birds and human.

## Supplementary Information

**Additional file 1: Table S1.** Ingredient and chemical composition of the experimental diets in chronic lipopolysaccharide challenge trial. **Table S2.** Ingredient and chemical composition of the experimental diets in acute lipopolysaccharide challenge trial. **Table S3.** The primers for quantitative real-time PCR.

**Additional file 2: Fig. S1.** Schematic presentation of **(A, B)** the experimental design. **(A)** Chronic LPS challenge route: ducks were fed basal diet or resistant starch (RS) diet for 18 d and intraperitoneally injected with either 1 mg/kg body weight (BW) of LPS or sterile saline at 14, 16, and 18 days of age. **(B)** Acute LPS challenge route: ducks were fed basal diet or resistant starch (RS) diet for 14 d and intraperitoneally injected with either 2 mg/kg body weight (BW) of LPS or sterile saline at 14 days of age. Besides, liraglutide (100 μg/kg BW) was injected at the same time as LPS injection in liraglutide group ducks. Both of chronic and acute LPS-challenge, at 4 h after injecting, 1 duck with a weight closest to the pen average was selected for samples collection. **(C)** the effect of dietary RS supplementation and liraglutide administration on body weight (BW) in ducks under acute LPS challenge.

## Abbreviations

DSS: Dextran sulfate sodium
GLP-1: Glucagon-like peptide-1
GLP-1R: Glucagon-like peptide 1 receptor
IFN-γ: Interferon-γ
IL: Interleukin
LPS: Lipopolysaccharide
Pcsk1: Proprotein convertase subtilisin/kexin type
RPS: Raw potato starch
RS: Resistant starch
SCFAs: Short chain fatty acids
Slc5al: Solute carrier family 5 member 1
TJPs: Tight junction proteins
TLR4: Toll like receptor 4
TLRs: Toll like receptors
TNF-α: Tumor necrosis factor alpha
