# Supplementary material for: Dietary resistant starch ameliorating lipopolysaccharide-induced inflammation in meat ducks associated with the alteration in gut microbiome and glucagon-like peptide 1 signaling
Source: J Anim Sci Biotechnol. 2022 Jul 15;13:91. doi: 10.1186/s40104-022-00735-x (PMC9284752; doi:10.1186/s40104-022-00735-x)
Supplement: Supplementary file 1 — Additional file 1: Table S1. Ingredient and chemical composition of the experimental diets in chronic lipopolysaccharide challenge trial. Table S2. Ingredient and chemical composition of the experimental diets in acute lipopolysaccharide challenge trial. Table S3. The primers for quantitative real-time PCR. [file 40104_2022_735_MOESM1_ESM.docx]

**Table S1.** Composition and nutrition level of the experimental diets (chronic LPS challenge, dry matter basis)

| Items | Basal diet | RS diet |
| --- | --- | --- |
| Ingredients, % |  |  |
| Corn | 59.82 | 44.85 |
| Soybean meal | 33.22 | 35.91 |
| Raw potato starch | 0.00 | 12.00 |
| Soybean oil | 0.50 | 1.50 |
| Calcium Carbonate | 1.10 | 1.04 |
| Dicalcium phosphate | 1.75 | 1.82 |
| L-Lysine·HCL | 0.12 | 0.07 |
| DL-Methionine | 0.16 | 0.17 |
| L-Threonine | 0.02 | 0.02 |
| Bentonite | 2.33 | 1.64 |
| Sodium chloride | 0.30 | 0.30 |
| Choline chloride | 0.15 | 0.15 |
| Vitamin premix^1^ | 0.03 | 0.03 |
| Mineral premix^2^ | 0.50 | 0.50 |
| Total | 100 | 100 |
| Calculated nutrient analysis, % | | |
| AME Kcal/kg | 2800 | 2800 |
| CP | 19.50 | 19.50 |
| Ca | 0.90 | 0.90 |
| AP | 0.42 | 0.42 |
| Dig. Lys | 1.00 | 1.00 |
| Dig. Met | 0.43 | 0.43 |

AME = apparent metabolizable energy; CP = crude protein; Ca = calcium; AP = available phosphorus; Dig. = digestibility.

^1^Provided per kg of diet: vitamin A, 12,000 IU; vitamin D_3_, 3000 IU; vitamin E, 7.5 mg; thiamine, 0.6 mg; riboflavin, 4.8 mg; pyridoxine, 1.8 mg; vitamin B_12_, 0.01 mg; calcium pantothenate, 7.5 mg; folate, 0.15 mg; niacin, 10.5 mg.

^2^Provided per kg of diet: Cu (CuSO_4_∙5H_2_O), 8 mg; Fe (FeSO_4_∙7H_2_O), 60 mg; Zn (ZnSO_4_∙7H_2_O), 60 mg; Mn (MnSO_4_∙H_2_O), 100 mg; Se (NaSeO_3_), 0.3 mg; I (KI), 0.4 mg.

**Table S2.** Ingredient and chemical composition of the experimental diets (acute LPS challenge, dry matter basis)

| Items | Basal | RS diet |
| --- | --- | --- |
| Ingredients, % |  |  |
| Corn | 59.82 | 45.00 |
| Soybean oil | 1.72 | 2.65 |
| Soybean meal | 33.22 | 35.90 |
| Raw potato starch | 0.00 | 12.00 |
| L-Lysine·HCl | 0.11 | 0.06 |
| DL-methionine | 0.157 | 0.166 |
| Threonine | 0.015 | 0.013 |
| Limestone | 1.10 | 1.05 |
| Dicalcium phosphate | 1.75 | 1.85 |
| Medical stone | 1.128 | 0.331 |
| Sodium chloride | 0.30 | 0.30 |
| Choline chloride | 0.15 | 0.15 |
| Vitamin premix^1^ | 0.03 | 0.03 |
| Mineral premix^2^ | 0.50 | 0.50 |
| Total | 100 | 100 |
| Calculated nutrient level, % |  |  |
| AME, Kcal/kg | 2900 | 2900 |
| CP | 19.50 | 19.50 |
| Ca | 0.90 | 0.90 |
| AP | 0.42 | 0.42 |
| Dig. Lys | 1.00 | 1.00 |
| Dig. Met | 0.43 | 0.43 |

AME = apparent metabolizable energy; CP = crude protein; Ca = calcium; AP = available phosphorus; Dig. = digestibility.

^1^Provided per kg of diet: vitamin A, 12,000 IU; vitamin D_3_, 3000 IU; vitamin E, 7.5 mg; thiamine, 0.6 mg; riboflavin, 4.8 mg; pyridoxine, 1.8 mg; vitamin B_12_, 0.01 mg; calcium pantothenate, 7.5 mg; folate, 0.15 mg; niacin, 10.5 mg.

^2^Provided per kg of diet: Cu (CuSO_4_∙5H_2_O), 8 mg; Fe (FeSO_4_∙7H_2_O), 60 mg; Zn (ZnSO_4_∙7H_2_O), 60 mg; Mn (MnSO_4_∙H_2_O), 100 mg; Se (NaSeO_3_), 0.3 mg; I (KI), 0.4 mg

**Table S3.** The primers for quantitative real-time PCR.

| Gene | Gene ID | Primer | Sequence (5′-3′) | Size, bp |
| --- | --- | --- | --- | --- |
| *Occludin* | XM_013109403.1 | Forward | aagcgctacaagcaggatgt | 147 |
|  |  | Reverse | cttgtcgtagtcgctcacca |  |
| *ZO-1* | XM_038184899.1 | Forward | tacgcctgtgaagaatgcag | 86 |
|  |  | Reverse | ggagtggtggtgtttgcttt |  |
| *Claudin-1* | XM_013108556.4 | Forward | ttggaatcctcttgggactg | 146 |
|  |  | Reverse | caaaacagcaagacctgcaa |  |
| *GLP-1R* | XM_013103405.4 | Forward | ttccaggaatccctcatctg | 95 |
|  |  | Reverse | aggtacatgccttccaccag |  |
| *Pcsk1* | XM_027446812.2 | Forward | tctggaggcaaatccaaatc | 114 |
|  |  | Reverse | agtcccgctccattcttttt |  |
| *Slc5a1* | XM_005026696.5 | Forward | tcagcaaggaggaagaggaa | 127 |
|  |  | Reverse | aaggcatggcaaaatacagc |  |
| *TNF-α* | EU375296.1 | Forward | agatgggaagggaatgaacc | 51 |
|  |  | Reverse | gttggcataggctgtcctgt |  |
| *IFNγ* | NM_001310417.1 | Forward | actggcttgaaaatccaacg | 101 |
|  |  | Reverse | ggagactggctccttttcct |  |
| *IL-1β* | DQ393268.1 | Forward | gcatcaagggctacaagctc | 131 |
|  |  | Reverse | caggcggtagaagatgaagc |  |
| *IL-4* | XM_005024359.3 | Forward | ttttgcaggcaatgagacag | 123 |
|  |  | Reverse | acgatgtgcagcaagttgag |  |
| *IL-6* | XM_027450925.2 | Forward | ccagaaatccctcctcacaa | 110 |
|  |  | Reverse | ccctcacggttttctccata |  |
| *IL-17* | EU366165.1 | Forward | atgcctgacccaaaaagatg | 145 |
|  |  | Reverse | gtggtcctcatcgatcctgt |  |
| *IL-10* | NM_001310368.1 | Forward | ctgacctcctaccagcgaag | 106 |
|  |  | Reverse | gagctgagcagctgaatgc |  |
| *TLR4* | NM_001310413.1 | Forward | cagctgagtgtcctgttgga | 141 |
|  |  | Reverse | cagcaggtcctccttttctg |  |
| *β-actin* | NM_001310408.1 | Forward | ccagccatctttcttgggta | 105 |
|  |  | Reverse | gtgttggcgtacaggtcctt |  |
| *GAPDH* | XM_005016745.3 | Forward | tttttaaccgtggctccttg | 94 |
|  |  | Reverse | actgggcatggaagaacatc |  |

*ZO-1* = zonula occludens-1; *GLP-1R =* glucagon-like peptide-1 receptor; *Pcsk1 =* proprotein convertase subtilisin/kexin type 1; *Slc5a1 =* solute carrier family 5 member 1; *TNF-α* = tumor necrosis factor-α; *IFNγ* = Interferon gamma; *IL =* interleukin; *TLR4 =* toll-like receptor 4; *GAPDH* = glyceraldehyde-3-phosphate dehydrogenase.


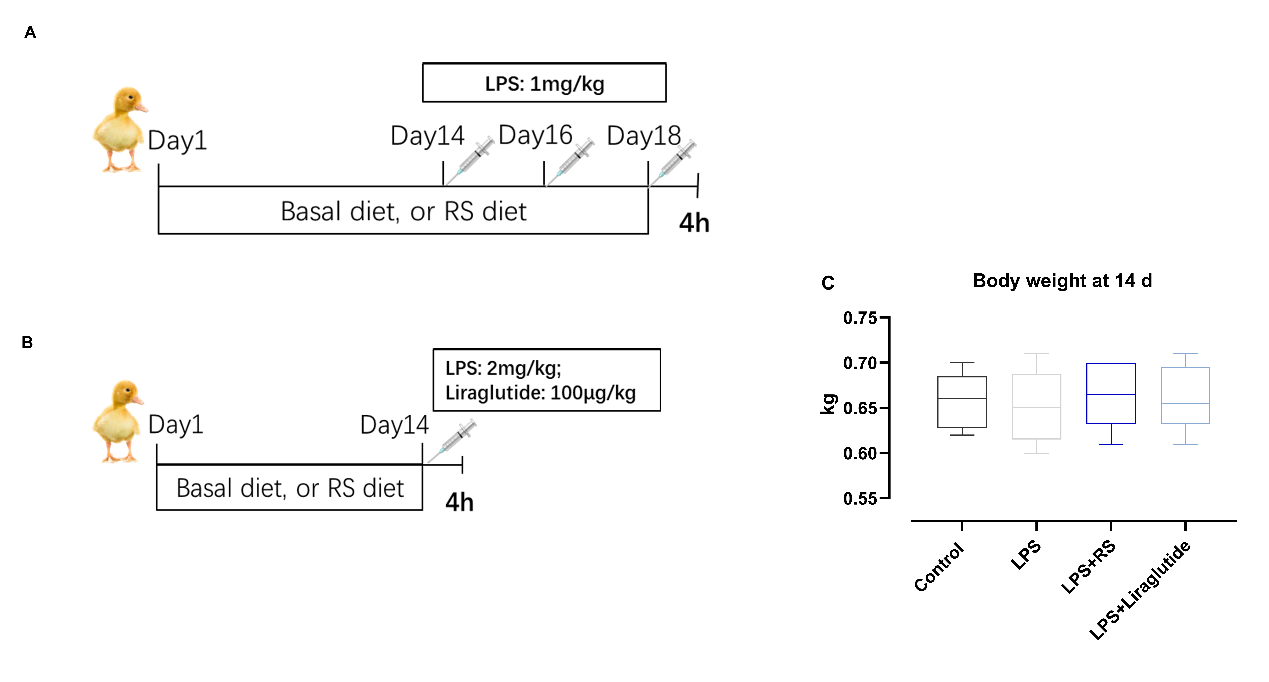


**Fig. S1.** Schematic presentation of **(A, B)** the experimental design and **(C)** the effect of dietary RS supplementation and liraglutide administration on body weight (BW) in ducks under acute LPS challenge.
