# Supplementary figures and images for: Dietary resistant starch ameliorating lipopolysaccharide-induced inflammation in meat ducks associated with the alteration in gut microbiome and glucagon-like peptide 1 signaling
Source: J Anim Sci Biotechnol. 2022 Jul 15;13:91. doi: 10.1186/s40104-022-00735-x (PMC9284752; doi:10.1186/s40104-022-00735-x)

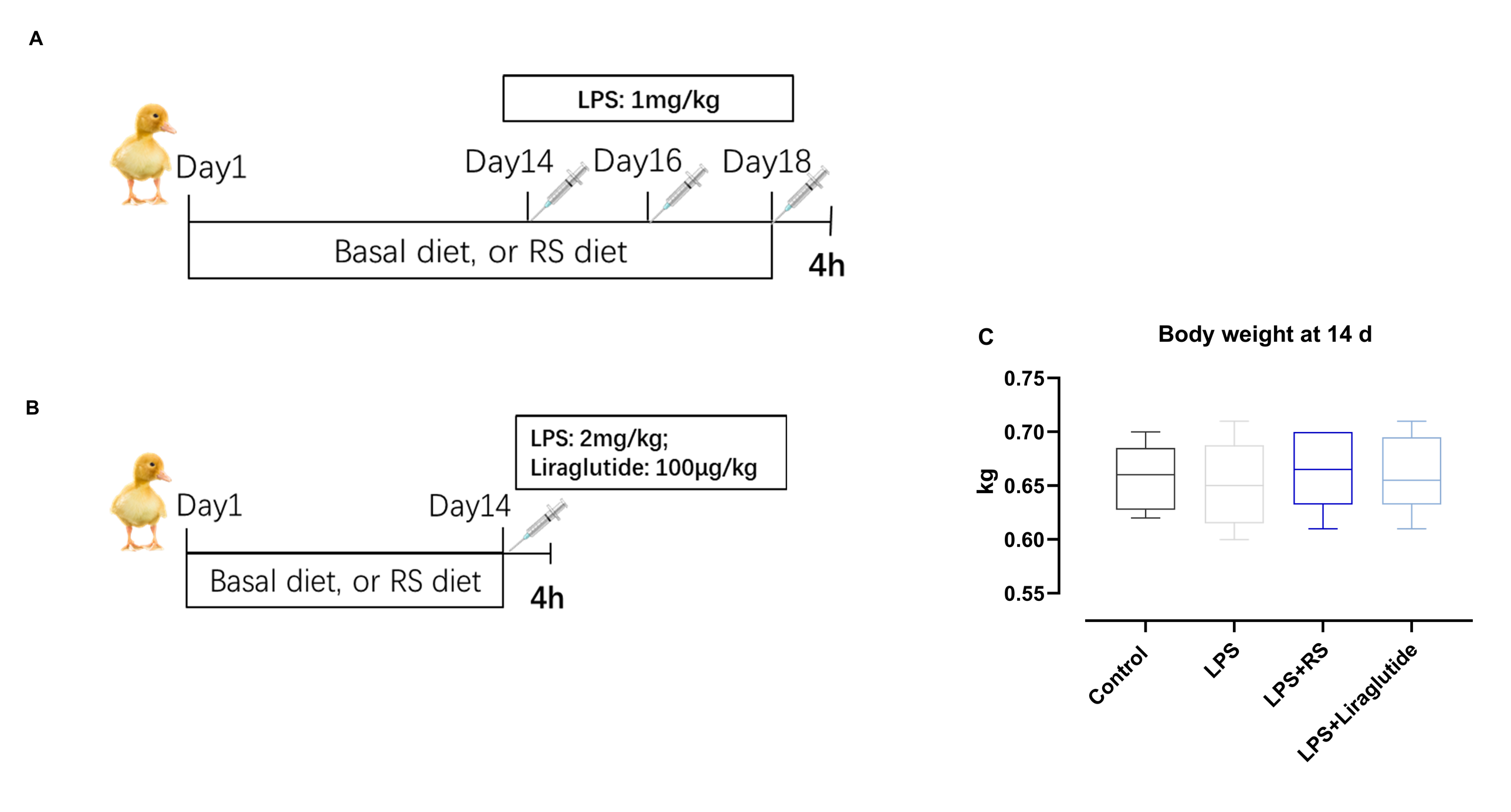

Supplement: Supplementary file 2 — Additional file 2: Fig. S1. Schematic presentation of (A, B) the experimental design. (A) Chronic LPS challenge route: ducks were fed basal diet or resistant starch (RS) diet for 18 d and intraperitoneally injected with either 1 mg/kg body weight (BW) of LPS or sterile saline at 14, 16, and 18 days of age. (B) Acute LPS challenge route: ducks were fed basal diet or resistant starch (RS) diet for 14 d and intraperitoneally injected with either 2 mg/kg body weight (BW) of LPS or sterile saline at 14 days of age. Besides, liraglutide (100 μg/kg BW) was injected at the same time as LPS injection in liraglutide group ducks. Both of chronic and acute LPS-challenge, at 4 h after injecting, 1 duck with a weight closest to the pen average was selected for samples collection. (C) the effect of dietary RS supplementation and liraglutide administration on body weight (BW) in ducks under acute LPS challenge. [file 40104_2022_735_MOESM2_ESM.tif]
